# The relationship with clinical course and prognosis of serum endothelin-1, angiopoietin-2, and tie-2 levels in Crimean-Congo hemorrhagic fever

**DOI:** 10.3906/sag-1812-10

**Published:** 2019-08-08

**Authors:** Ferhan KERGET, Zülal ÖZKURT, Nurinnisa ÖZTÜRK, Sinan YILMAZ

**Affiliations:** 1 Department of Infection Diseases and Clinical Microbiology, University of Health Sciences, Erzurum Regional Education and Research Hospital, Erzurum Turkey; 2 Department of Infection Diseases and Clinical Microbiology, School of Medicine, Atatürk University, Erzurum Turkey; 3 Department of Biochemistry, School of Medicine, Atatürk University, Erzurum Turkey; 4 Department of Public Health, Atatürk University, School of Medicine, Erzurum Turkey

**Keywords:** Angiopoietin-2, endothelin-1, Crimean-Congo hemorrhagic fever, Tie-2

## Abstract

**Background/aim:**

Crimean-Congo hemorrhagic fever (CCHF) is a serious illness characterized by fever and hemorrhage. Endothelin-1 (ET-1), angiopoietin-2 (Ang-2), and endothelial cell-specific receptor tyrosine kinase (Tie-2) are believed to be important markers of the pathogenesis, clinical course, and prognosis of the disease. The aim of this study was to determine ET-1, Ang-2, and Tie-2 levels in adults with CCHF and investigate the associations between these markers and pathogenesis and disease course.

**Materials and methods:**

Sixty CCHF patients were included in the study. The patients were classified according to disease severity criteria and Ang-2, Tie-2, and ET-1 levels were compared.

**Results:**

Mean serum ET-1 level was 36.62 ± 27.99 pg/mL in the patient group and 3.70 ± 4.71 pg/mL in the control group (P = 0.001). Mean serum Ang-2 levels were 2511.18 ± 1018.64 pg/mL in the patient group and 3570.76 ± 209.52 pg/mL in the control group (P = 0.001). Mean serum Tie-2 levels were 7.35 ± 7.75 ng/mL in the patient group and 0.67 ± 1.26 ng/mL in the control group (P = 0.001).

**Conclusion:**

Elevated ET-1 and Tie-2 levels were associated with more severe disease course, while Ang-2 level was negatively correlated with severity in adult CCHF patients. ET-1, Tie-2, and Ang-2 levels are important prognostic parameters in CCHF and may contribute significantly to treatment and follow-up.

## 1. Introduction

Crimean-Congo hemorrhagic fever (CCHF) is a serious illness that occurs each year in spring and summer in endemic regions. It presents with fever and hemorrhage, and in severe cases can lead to shock and death. The coagulation and fibrinolysis that develop in CCHF manifest clinically as petechiae, ecchymosis, mucosal hemorrhage, and uncontrollable hemorrhage at venous puncture sites. Monocytes, endothelial cells, and hepatocytes are the main target cells in CCHF. Macrophage activation and hemophagocytosis are likely pathological processes. The resulting vascular damage induces clotting dysfunction and increased capillary permeability, which result in hemorrhagic tendency. Inflammatory mediators play an important role in fatal cases [1,2].

Endothelin-1 (ET-1) is a key inflammatory mediator in abnormal vascular tone, vascular remodeling, and especially endothelial dysfunction [3]. Although present at very low levels in healthy individuals, studies have shown that ET-1 levels are high and correlated with clinical course in patients with diseases characterized by vascular and endothelial dysfunction such as CCHF [4].

Angiopoietin plays an important role in vascular stabilization and is stored in the Weibel–Palade bodies, which are exposed during endothelial damage. Four angiopoietins have been identified (Ang-1, -2, -3, and -4), and the most is known about Ang-1 and Ang-2. Ang-2 is released after endothelial damage and inhibits the action of Ang-1 by binding to its receptor, endothelial cell-specific receptor tyrosine kinase (Tie-2). It sensitizes the endothelium to inflammatory agents (vascular destabilization) and facilitates VEGF-mediated angiogenesis [5]. In a study of children with CCHF, Ang-2 and Tie-2 levels were observed to be elevated in correlation with disease course [6]. However, these parameters have not been analyzed in adult CCHF patients.

Therefore, this study was conducted to investigate whether ET-1, Ang-2, and Tie-2 levels are associated with mortality and disease course in adult CCHF patients with significant endothelial and vascular damage.

## 2. Materials and methods

### 2.1. Patient selection 

The study included 60 patients who were treated for CCHF in the Infectious Diseases and Clinical Microbiology Unit of the Atatürk University Hospital between March 2016 and September 2017. Fifteen healthy males and 15 females engaged in farming in and around Erzurum were included in the control group. Exclusion criteria for patients and healthy volunteers included the presence of known hepatic, renal, or pancreatic failure; gastrointestinal disease; acute cardiovascular failure; cerebrovascular disorders and hyperthyroidism; acute infections; pregnancy; and the use of antiinflammatory drugs within the last month. 

### 2.2. Study groups

The patients were divided into those with mild-moderate disease and those with severe disease according to the criteria defined by Swanepoel et al. and the modified criteria recommended by Ergönül et al. [7,8], as well as the clinical indicators of poor prognosis. Forty patients were included in the mild-moderate group, 16 patients in the severe group, and 4 patients in the fatal group.

### 2.3. CCHF diagnosis 

Two blood samples of at least 2 mL each were collected upon hospital admission from every patient with suspected CCHF. The samples were allowed to coagulate for 30 min and then separated by centrifugation at 2000 rpm for 5 min, and the serum was transferred to Eppendorf tubes. One serum sample from each patient was sent by appropriate transport methods to the Erzurum Regional Public Health Laboratory, which is the regional reference center, for serological and virological tests. The diagnosis of CCHF was based on specific anti-IgM antibody and/or polymerase chain reaction (PCR) positivity detected by immunofluorescence assay (IFA).

### 2.4. Assessment of serum ET-1, Ang-2, and Tie-2 levels

Venous blood samples collected from the patient and control groups were placed in serum tubes containing separator gel and centrifuged at 3500 rpm for 7 min. ET-1, Ang-2, and Tie-2 concentrations were analyzed using ELISA kits according to the manufacturer’s instructions (Elabscience, Wuhan, Hubei, China).

### 2.5. Statistical analysis

SPSS 20.0 (IBM Corp., Armonk, NY, USA) was used to analyze the study data. The Kolmogorov–Smirnov test was used to determine whether the data were normally distributed. Demographic characteristics, physical examination findings, and symptom frequency of the patients were also analyzed. Intergroup comparisons were done using a t-test for independent groups and the Mann–Whitney U test for data that were not normally distributed. The chi-square test was used to analyze categorical data, and the relationships between cytokines and other laboratory variables were investigated with Spearman correlation analysis for data that did not conform to normal distribution. The diagnostic value of ET-1, Ang-2, and Tie-2 was assessed using the receiver operating characteristic curve method and a cut-off point was determined for ET-1. Results with P < 0.05 were considered statistically significant.

## 3. Results

The patient group comprised 30 males (50%) and 30 females (50%). There were 18 males (60%) and 12 females (40%) in the control group. The mean ages of the patient and control groups were 48.93 ± 17.30 years and 42.27 ± 15.17 years, respectively. The differences in age and sex distribution between the groups were nonsignificant. Based on severity criteria, 16 (26.6%) patients were in the severe group and 40 (66.6%) patients were in the mild-moderate group, while 4 patients (6.8%) died. Of these, 3 died due to multiple organ failure, while the other patient developed chest pain, shortness of breath, and sudden loss of consciousness followed by cardiopulmonary arrest. The remaining 56 patients (93.2%) were discharged with full recovery. Thirty-five (58.3%) patients had a history of tick contact, while the other 25 (41.7%) patients were not aware of any tick contact. Forty-five (75%) patients had contact with animals, while the other 15 (25%) patients did not. The mean incubation period among patients with known history of tick contact was 3.45 days.

The most common symptoms were fatigue (100%), anorexia (100%), and fever (91.7%). Twenty-one patients (35%) exhibited hemorrhage either at admission or during follow-up, most commonly in the form of gingival (15%) and nasal bleeding (15%). Three patients (5%) had melena and 5 patients (8.3%) had vaginal bleeding.

The most common findings on physical examination were conjunctival hyperemia (98.3%), oropharyngeal mucosal hyperemia (93.3%), and rash (93.3%). Hemorrhage occurred in 21 patients (35%) during follow-up. Four patients (6.7%) developed bradycardia. One patient developed atrial fibrillation and later died.

The patients’ laboratory results at time of admission (Table 1) together with clinical indicators of poor prognosis were used to classify the patients as having mild-moderate or severe CCHF. 

**Table 1:** Analysis of laboratory parameters of CCHF patients.

Laboratory parameters	Mean (min–max)
Sedimentation (mm/h)	16.2 ± 17.6 (2–93)
CRP (mg/L)	26 ± 30.5 (3–116)
Hemoglobin (g/dL)	13.2 ± 2.4 (5–17)
WBC (103/µL)	2423.9 ± 1453.4 (390–8690)
Platelets (103/µL)	49,400 ± 37,465.1 (6000–141,000)
ALT (U/L)	280.2 ± 399.3 (17–2812)
AST (U/L)	713.5 ± 1644.2 (34–12090)
Creatine kinase (U/L)	898.1 ± 1396 (54–7940)
LDH(U/L)	1274 ± 1909 (238–12882)
BUN (mg/dL)	15.5 ± 9.9 (4–55)
Creatine (mg/dL)	0.9 ± 0.4 (0–3)
Glucose (mg/dL)	129.8 ± 47.9 (83–398)
PT (s)	13.8 ± 3 (10–24)
PTT (s)	40.4 ± 17.2 (20–113)
INR	1.1 ± 0.3 (1–2)

WBC: White blood cell count, ALT: alanine aminotransferase, AST: aspartate aminotransferase, LDH: lactate dehydrogenase, BUN: blood urea nitrogen, PT: prothrombin time, PTT: partial thromboplastin time, INR: international normalized ratio.

Mean ET-1 levels were 27.97 ± 16.21 pg/mL in mild and 43.47 ± 28.26 pg/mL in severe cases (P = 0.03). Mean Ang-2 levels were 2836.06 ± 776.76 pg/mL in mild and 2173.51 ± 1057.25 pg/mL in severe cases (P = 0.01). Mean Tie-2 levels were 4.54 ± 4.55 ng/mL in mild and 13.86 ± 1.26 ng/mL in severe cases (P = 0.001). Comparison of serum ET-1, Ang-2, and Tie-2 levels of the severe patients group and the healthy control group revealed highly significant differences (Table 2).

**Table 2:** Comparison of serum ET-1, Ang-2, and Tie-2 levels of patients and healthy control group.

Groups	ET-1 (pg/mL)	Ang-2 (pg/mL)	Tie-2 (ng/mL)
Mild-moderate patients	28 ± 16.2	2836.1 ± 776.8	4.5 ± 4.6
Severe patients	43.5 ± 28.3	2173.5 ± 1057.3	13.9 ± 1.3
Healthy controls	3.7 ± 4.7	3570.8 ± 209.5	0.7 ± 1.3
P*	0.001	0.001	0.001
P**	0.03	0.01	0.001

P*: Comparison of healthy controls and severe patients, P**: Comparison of mild-moderate and severe patients.

Highly significant differences in median serum ET-1, Ang-2, and Tie-2 values were observed between the patient and healthy control groups (Table 3).

**Table 3:** Comparison of serum ET-1, Ang-2, and Tie-2 levels of patients and healthy control group, and patients with and without hemorrhage in CCHF.

Groups	ET-1 (pg/mL)	Ang-2 (pg/mL)	Tie-2 (ng/mL)
Patients	36.6 ± 27.9	2511.2 ± 1018.6	7.4 ± 7.8
Controls	3.7 ± 4.7	3570.8 ± 209.5	0.7 ± 1.2
P	0.001	0.001	0.001
Hemorrhage			
Yes	33 ± 29.9	3005 ± 1118	10.4 ± 9.3
No	21 ± 26.5	3100 ± 943.3	3.5 ± 5.4
P	0.107	0.041	0.002

Evaluation of patients’ serum ET-1, Ang-2, and Tie-2 levels according to mortality showed that surviving patients had lower mean ET-1 and Tie-2 levels and higher mean Ang-2 levels compared to deceased patients (Table 4). However, the differences could not be evaluated statistically because the number of deceased patients was too small to use a nonparametric test.

**Table 4:** Laboratory parameters of deceased patient and surviving patients, with analysis of serum ET-1, Ang-2, and Tie-2 levels.

	Surviving patients (n = 56)(min–max)	Deceased patients (n = 4)(min–max)
Laboratory parameters		
ET-1 (pg/mL)	32.4 (11.6–101.8)	95.6 (39.7–150.7)
Ang-2 (pg/mL)	2646.7 (46.9–3431)	613.1 (84.4–1036.8)
Tie-2 (ng/mL)	7.2 (0.2–29.4)	9.4 (2.83–26.8)
WBC (103/µL)	2399.7 (390–8690)	2763.5 (1500–4330)
Platelets (103/µL)	51,464.3 (6000–141,000)	20,500 (16,000–31,000)
ALT (U/L)	210.8 (17–706)	1251.7 (270–2812)
AST (U/L)	412.5 (34–1975)	4927.2 (758–12,090)
Creatine kinase (U/L)	824.4 (54–7940)	1929.5 (442–4816)
LDH (U/L)	890.6 (238–3313)	6649.7 (1382–12,882)
BUN (mg/dL)	14.9 (4–55)	23.2 (10–39)
Creatine (mg/dL)	0.8 (0–3)	0.9 (0.6–1)
Glucose (mg/dL)	126.7 (83–398)	171.5 (102–265)
PT (s)	13.6 (10–23)	17 (11–24)
PTT (s)	38.7 (20–91)	64.5 (40–113)
INR	1.1 (1–2)	1.4 (1–2)

The patients’ serum ET-1, Ang-2, and Tie-2 levels were compared with the following laboratory values: white blood cell count (WBC), platelet count (PLT), aspartate aminotransferase (AST), alanine aminotransferase (ALT), creatine phosphokinase (CPK), lactate dehydrogenase (LDH), prothrombin time (PT), partial thromboplastin time (PTT), and international normalized ratio (INR). There was a weak but significant positive correlation between Ang-2 and PLT, whereas significant weak negative correlations were observed between Ang-2 and ALT, LDH, and PTT. ET-1 showed a weak negative correlation with Ang-2 and a weak positive correlation with Tie-2. There was a weak negative correlation between Tie-2 and PLT, and a weak positive correlation between Tie-2 and LDH (Table 5).

**Table 5:** The comparison of laboratory findings of CCHF patients for serum ET-1, Ang-2, and Tie-2 levels.

Laboratory parameters	PTT (r/p)	INR (r/p)	ET-1 (r/p)	Ang-2 (r/p)	TIE-2 (r/p)
WBC (103/µL)	0.031/0.814	0.105/0.425	0.181/0.186	–0.057/0.666	0.069/0.602
Platelets (103/µL)	–0.460/0.001	–0.129/0.327	–0.242/0.063	0.481/0.001	–0.281/0.029
ALT (U/L)	0.506/0.001	0.150/0.253	0.135/0.305	–0.393/0.002	0.116/0.377
AST (U/L)	0.636/0.001	0.187/0.152	0.168/0.199	–0.437/0.001	0.204/0.118
Creatine kinase (U/L)	0.532/0.001	0.312/0.015	0.03/0.820	–0.279/0.031	0.031/0.815
LDH (U/L)	0.654/0.001	0.201/0.123	0.220/0.092	–0.459/0.001	0.269/0.038
PT	0.231/0.076	0.818/0.001	–0.009/0.943	0.128/0.331	–0.125/0.343
PTT		0.446/0.001	0.166/0.206	–0.368/0.004	0.193/0.14
INR			0.046/0.730	0.141/0.281	–0.087/0.508
ET-1				–0.436/0.001	0.486/0.001
Ang-2					–0.226/0.083

WBC: White blood cell count, ALT: alanine aminotransferase, AST: aspartate aminotransferase, LDH: lactate dehydrogenase.

In terms of clinical findings, serum ET-1, Ang-2, and Tie-2 levels were compared in patients with and without hemorrhage and patients with and without lung auscultation findings. Significant differences in median Ang-2 and Tie-2 values were observed between patients with and without hemorrhage.

## 4. Discussion 

In this study, we determined that elevated serum ET-1 and Tie-2 levels were associated with mortality and clinical severity in patients with CCHF, while Ang-2 levels were negatively correlated with mortality and clinical course. 

CCHF manifests with sudden fever, weakness, diffuse myalgia and arthralgia, headache, and sore throat. These symptoms are sometimes accompanied by complaints of nausea, vomiting, abdominal pain, and watery diarrhea. Early signs may include hypotension, conjunctival hyperemia, and blanching macular rash [7,9].

As with all viral hemorrhagic fevers (VHFs), the most important fact known about the pathogenesis of CCHF is that the virus primarily infects endothelial cells, mononuclear cells, and hepatocytes, as well as many other cell types, and proliferates to spread systemically. Increased endothelial permeability and the resulting endothelial damage have been implicated as the main pathogenetic process leading to death.

Endothelial cells are among the main targets of the CCHF virus [10,11]. Endothelial damage leads to the activation of the intrinsic coagulation cascade, and the activation of platelet adhesion, aggregation, and degranulation results in hemostatic imbalance. Ultimately, it leads to disseminated intravascular coagulation (DIC) and diffuse hemorrhage due to excessive consumption of coagulation factors [12]. In addition, liver function impairment caused by the viral cytopathic effect in CCHF results in reduced synthesis of plasma coagulation factors, as most of these factors are synthesized in the liver [13]. During the follow-up of the 60 patients in our study, hemorrhage associated with DIC was observed in 16 patients (26.6%), and 4 of the patients (6.8%) died due to these hemorrhages and multiple organ failure. Although hemorrhage is considered the primary cause of mortality due to CCHF, multiple organ failure was observed to be the most important cause of death in our study. 

ET-1, one of the principal markers of endothelial cell dysfunction and vascular damage, is a 21-amino-acid vasoactive peptide that plays a role in vascular remodeling and the pathogenesis of many diseases. ET-1 is present in several vital organs of the body and interacts with transcription factors to stimulate expression of tumor necrosis factor (TNF)-alpha, interleukin (IL)-1, and IL-6. Inflammatory cytokines and angiogenic proteins are the key mediators in the maintenance of vascular integrity.

Vascular endothelial growth factor (VEGF) and angiopoietins (Ang) are the most important regulators of vascular integrity, especially in VHFs. The best known angiopoietins are Ang-1 and Ang-2. Ang-2 is almost exclusively produced in endothelial cells and stored in Weibel–Palade bodies, and it is released upon the activation of the endothelium. The effect of Ang-2 begins at the leukocyte adhesion step. It activates the endothelium and promotes the inflammatory response by increasing endothelial permeability. Thus, Ang-2 triggers the inflammatory response, impairs endothelial balance, and renders the endothelium susceptible to external influences. VEGF and TNF-alpha stimulate Ang-2 synthesis. VEGF levels have been shown to be lower in severe and fatal cases of CCHF [14–16]. In another study of CCHF patients, TNF-alpha levels were lower in severe cases [14]. These findings may explain the low Ang-2 levels in patients.

Like the Ang system, Tie-2 plays an important role in the pathogenesis of VHFs. Ang-1 activates the Tie-2 receptor when it binds, thus supporting vascular stabilization. By preventing the adhesion of circulating leukocytes to the endothelium, Tie-2 assumes the dynamic barrier function of the endothelium and enables the endothelium to remain quiet [17]. In contrast, Ang-2 is an antagonist and inactivates the Tie-2 receptor when it binds, resulting in vascular imbalance [18]. Ang-2 acts as a Tie-2 antagonist. Therefore, if Tie-2 is high, a low Ang-2 level can be expected. At the same time, Tie-2 also demonstrates the body’s strong defense against endothelial impairment.

Ang-2 and Tie-2 levels were previously found to be higher in pediatric CCHF patients compared to the healthy group and were correlated with clinical course and prognosis [6]. In another study, Ang-2 was shown to be associated with vascular damage and prognosis in patients infected with the dengue virus [19].

Although higher Ang-2 levels were reported in severe disease in some of the aforementioned studies, we observed lower levels in our study. There are several possible reasons for this finding. First, the virus itself may suppress the cellular immune response in infected monocytes and lymphocytes. A second possibility is that despite the expectation that early viremia will act as an immunogen and stimulate the immune system, it may cause immune tolerance and paralyze the immune system. Alternatively, as Ang-2 is synthesized exclusively by endothelial cells and severe cases have more endothelial damage, we believe that the synthesis of Ang-2, along with other endothelial functions, may be inadequate in these patients.

In conclusion, elevated ET-1 and Tie-2 levels are closely associated with poor prognosis and mortality in CCHF. On the other hand, Ang-2 level was negatively correlated with clinical course and mortality. ET-1, Tie-2, and Ang-2 levels may provide guidance in terms of disease course and clinical follow-up. Multiple organ failure was the main cause of death due to CCHF in our study. More extensive studies evaluating viral load and other constituents of the angiopoietic system together are needed to reach more definitive conclusions about this subject.

## Compliance with ethical standards

**Ethical statement:** This study was approved by the Atatürk University Ethics Committee (30.11.2016, 8/88) and has been performed in accordance with the ethical standards laid down in the 1964 Declaration of Helsinki and its later amendments or comparable ethical standards. 
